# Meat consumption and obesity: A climate‐friendly way to reduce health inequalities

**DOI:** 10.1002/puh2.163

**Published:** 2024-03-15

**Authors:** Laura Sares‐Jäske, Heli Tapanainen, Liisa Valsta, Peppi Haario, Satu Männistö, Maria Vaalavuo

**Affiliations:** ^1^ Finnish Institute for Health and Welfare Helsinki Finland

**Keywords:** climate impact, education, health inequalities, lifestyle habits, obesity, processed meat, red meat

## Abstract

**Background:**

Climate change, health inequalities and obesity are considerable public health challenges of the 21st century. Red and processed meat (RPM) consumption is associated with an increased risk of obesity and with higher climate impact. At the same time, educational inequalities exist not only in RPM consumption and obesity but also in other health behaviours. Thus, we investigated whether educational inequalities exist in the association between RPM consumption and obesity, while also considering health behaviours (physical activity, vegetable, legume and fruit consumption, alcohol consumption and smoking) as potential confounding and effect modifying factors.

**Methods:**

The FinHealth 2017 Study data, including 4494 participants aged 18–74 years, were used. A validated food frequency questionnaire was employed to determine dietary intake. Height and weight were measured by trained study nurses. Linear and logistic regression models were used.

**Results:**

Odds of obesity increased along with RPM consumption in women (*p* < 0.001) and men (*p* < 0.001) and in each educational group regardless of other unfavourable health behaviours. Only in men with basic education were the differences between RPM consumption categories not statistically significant. Compared to those with high education and the lowest RPM consumption, those with basic education and the highest RPM consumption had multiple odds of obesity (odds ratio (95% confidence interval) among women: 7.5 (2.7–20.4); among men: 5.3 (2.5–11.1)).

**Conclusion:**

High RPM consumption appears to be associated with obesity independently of other unfavourable health behaviours or education, yet the odds are higher with basic education. Targeting unhealthy dietary patterns with heavy ecological burden could help reduce both health inequalities and mitigate climate change.

## Graphical abstract

Even though associations between red and processed meat (RPM) consumption, obesity, education and unfavourable lifestyle habits are complex, high RPM consumption appears to be associated with obesity independently of other unfavourable health behaviours or education.

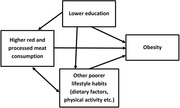

## INTRODUCTION

Climate change, health inequalities and obesity are considerable public health challenges of the 21st century. Several studies have demonstrated that a shift towards more plant‐based diets is needed to reduce greenhouse gas emissions [1, 2, 3]. Animal‐based foods generally have greater environmental impact than plant‐based foods, and of them, red meat, mostly comprising ruminant meat, creates the greatest climate burden [4]. In addition to the high environmental burden, the consumption of red and processed meat (RPM) also contributes to poorer health, as it has been shown to have a strong positive association with obesity and several other chronic diseases at the population level [5, 6, 7, 8, 9]. Various meta‐analysis studies have concluded that a shift towards more plant‐based diets that provide nutrients closer to recommended levels, while decreasing consumption of RPM, could decrease the risk of diet‐related health problems and diseases, such as diabetes, cardiovascular diseases, and several cancers [2, 10–12]. The World Health Organization summarised that while red meat can be an important element of a healthy diet at specific life stages by providing essential nutrients, excess consumption of RPM is associated with an increased risk of noncommunicable diseases, and many forms of livestock farming are associated with unsustainable environmental impacts [13]. In addition, the new Nordic Nutrition Recommendations 2023, based on a comprehensive literature review on health and climate effects of diet, concluded that RPM consumption should be considerably decreased both for health and environmental reasons [14].

There is a strong and persisting association between health and socio‐economic status (SES), regardless of the health indicator used or the definition of SES [15, 16, 17, 18]; individuals with higher income and educational level tend to have better health status than those with lower income and low education. Despite the predominantly favourable development in population health in Finland, inequalities in health behaviours and health indicators seem to persist and, in some cases, even widen [19]. In 2017, for example, less than one quarter of Finnish men and women with high education had obesity, whereas among those with basic education, the corresponding share was approximately one third [20]. Between years 2017 and 2023, the prevalence of the obesity in the working age Finnish population grew, whereas differences between the educational groups remained [21]. An inverse gradient between education and adiposity has also been found in other high‐income countries [22].

Unhealthy lifestyle habits, such as lack of physical activity (PA), unhealthy diet, alcohol risk use, and smoking, are largely responsible for the incidence of many noncommunicable diseases and premature deaths [23, 24, 25]. Lifestyle habits have been shown to accumulate so that individuals with one unfavourable habit also tend to have other unfavourable habits [26, 27, 28, 29]. Individuals with lower SES often have poorer lifestyle habits [30, 31] and consequently, lifestyle factors are considered important in accounting for health and obesity differences [32]. Generally, reasons for socio‐economic inequalities in health behaviours and unhealthy habits in individuals of lower SES have been suggested to include using unhealthy habits as ‘self‐medication’ in stressful everyday life, not finding that one would benefit from healthy lifestyle choices or not having enough information on them, lack of self‐efficacy and intentional class distinction between SES groups [31]. It is possible that differences in wealth, residential areas, nature of work and social relationships, for example, are drivers for choosing differing lifestyle habits. Motives behind food selection among individuals with lower education tend to be more based on lower price and familiarity and not on health or ethical concerns [33], which could also explain higher consumption of RPM among individuals with lower SES. Globally, food prices affect consumers’ consumption habits and have the greatest impact on households with low income [34]. In the United States and Europe, researchers have suggested that the price of healthy food and inequalities in access to it can explain higher rates of obesity and diabetes among lower SES groups [35, 36, 37].

**FIGURE 1:**
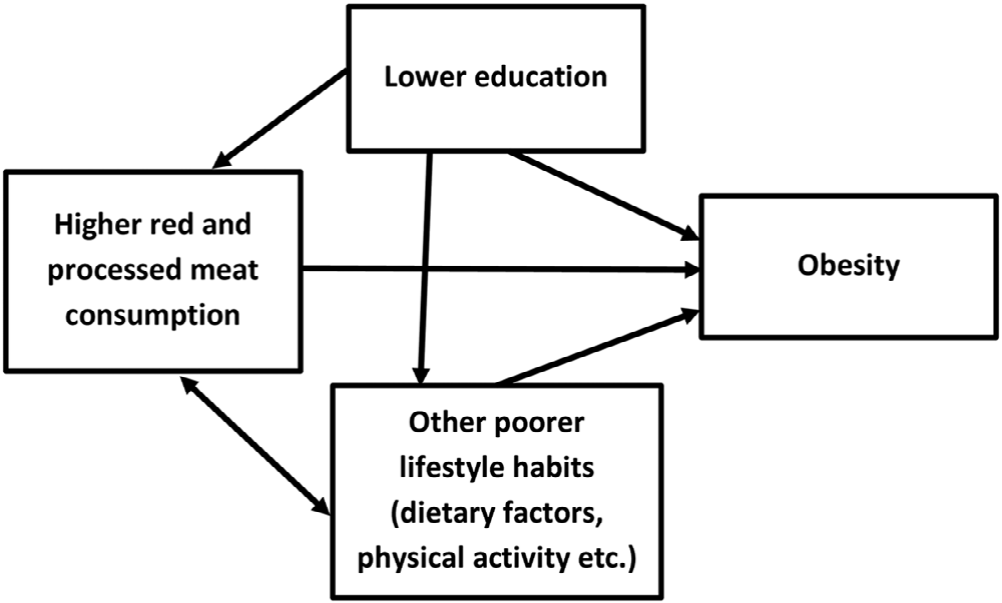
Associations between red and processed meat (RPM) consumption, odds of obesity, education and unfavourable lifestyle habits.

Associations between RPM consumption, obesity, education and unfavourable lifestyle habits are complex (Figure 1); higher RPM consumption, lower education and unhealthy lifestyle habits are related to increased risk of obesity, but also to each other. Thus, it is preferable to take these factors into account simultaneously when examining the associations between RPM consumption and obesity. In addition, due to significant sex differences in lifestyle habits, including RPM consumption [38], and sustainability of consumer preferences [39], it is reasonable to study the associations separately in women and men.

Our principal research question was whether the association between unhealthy and unecological food consumption and obesity varies across educational groups. Second, we studied to what extent the associations are explained by the accumulation of other unhealthy behaviours. Further, to broaden the aspect of SES, we studied whether the association between RPM consumption and obesity varies across income groups.

## METHODS

### Study design, data collection and participants

This study utilised the nationally representative FinHealth 2017 Study [40]. Data were collected employing health examinations (including measurements and drawing blood samples), questionnaires and interviews.

The invited sample comprised 10,247 individuals aged 18 and older. A total of 5952 (58%) individuals participated in the health examination, and 5125 filled in the food frequency questionnaire (FFQ). Of those participants, we excluded those aged 75 years or older (*n* = 556), those who were pregnant during the examination (*n* = 30), and those who had missing information on sex (*n* = 0), body mass index (BMI) (*n* = 94) or education (*n* = 0), comprising a sample of 4494 individuals. Further, of that study sample, the following numbers of individuals had missing information in the following variables that were used as confounders or as effect‐modifying factors: residential area *n* = 1, household structure *n* = 16, employment status *n* = 9, household income *n* = 134, leisure‐time PA *n* = 37, alcohol consumption *n* = 21 and smoking *n* = 31.

### Exposures, outcomes and other covariates

RPM, VLF (vegetable, legume and fruit) and energy intakes were determined with a validated [41, 42, 43] semi‐quantitative FFQ containing 134 foods, food groups and beverages, and measuring habitual food intake over the last 12 months [40, 44]. Ten frequency options ranging from ‘none’ to ‘6+ times a day’ were provided. Specific food groups and energy intakes were calculated using in‐house dietary software (Finessi) [45] based on the national food composition database (Fineli). RPM consumption was determined as the intake of red meat and processed meat (beef, pork, lamb, game, offal, sausages, sausage cuts and cold cuts) as grammes (g) per day. VLF consumption was determined as intake of vegetables, mushrooms, legumes, legume products, fruit and berries (excluding juices, canned fruit and vegetables and liquid legume products) as grammes (g) per day. Further, RPM and VLF consumptions were adjusted for total energy intake by using the residual method [46] to obtain the relative amount of these ingredient groups’ consumption in diet, regardless of energy intake. Further, energy‐adjusted RPM and VLF variables were divided into sex‐specific quintiles. Additionally, for the lifestyle‐interaction analyses, energy‐adjusted RPM variable was divided into sex‐specific tertiles.

Height and weight were measured during a health examination by trained nurses [40]. Height was measured with a stand‐alone stadiometer, with the participant standing upright without shoes [47]. Weight was measured with a Tanita bioimpedance analysis device in indoor clothes, automatically reducing 0.5 kg for the clothes. Obesity was determined as BMI ≥ 30 kg/m^2^.

Education was chosen to serve as an effect‐modifying factor in the analyses. Information on education, based on the highest completed degree, was drawn from national registers of Statistics Finland. Education was categorised into three classes: (1) basic (comprehensive school [years 1–9 in the current school system in Finland] or lower), (2) intermediate (upper secondary school [high school] or vocational school [usually 3–4 years after comprehensive school]) and (3) high (lower or higher university degree, university of applied sciences degree, polytechnic degree or higher).

Age, residential area, household structure, employment status and household income were chosen to serve as sociodemographic and socio‐economic confounding factors. Information on sex, age (categorised into groups of 18–34/35–54/55–74 years) and residential area (categorised into urban areas/areas near urban areas and rural centres/remote rural areas) was obtained from the sampling frame and originally from the Population Register Centre.

For other confounding factors, information was collected using questionnaires. Household structure was categorised as follows: household with only one adult living alone/household with at least one adult and at least one underage child/household with at least two adults and no underage children. Employment status was categorised into employed (including entrepreneurs and those working for a family business without salary)/other (including students, those retired, unemployed, on family‐leave and others). The income variable was based on questions on total household income during the last year before tax deductions and on the number of adult and underage household members. The household income variable consisted of 10 predefined categories from ‘less than €15,000’, and ‘€15,001–€25,000’ to ‘more than €90,000’. In this study, upper limits of the categories (and in the highest category, lower limit multiplied by two) were divided by the weighted sum of household members, given a value of 1.0 to the first adult, a value of 0.7 to additional adults and a value of 0.5 to the underage household members [48]. The quotient was further categorised into sex‐specific quintiles. For sensitivity analyses examining the association between RPM consumption and obesity in categories stratified by income, household income was also categorised into sex‐specific quartiles and further into three categories, in which case the two middle quartiles were combined.

Low leisure‐time PA, low VLF consumption, risk use of alcohol and daily smoking were chosen to represent unfavourable lifestyle. A four‐category question tracing leisure‐time PA was classified into a binary variable: low PA/moderate or strenuous PA or a competing athlete. The lowest energy‐adjusted VLF‐consumption quintile denoted low VLF consumption. Information on alcohol consumption was enquired with questions belonging to the Alcohol Use Disorders Identification Test, and risk use was defined separately for men and women (see more detailed information in Ref. [40]). Information concerning smoking was collected with a set of questions (see more detailed information in Ref. [40]), and a five‐category variable was formed. For this study, smoking was further categorised into daily smokers/others.

Accumulation of unfavourable lifestyle habits was determined by combining four two‐category lifestyle variables into a sum variable, with the unfavourable category of each variable scoring 1 and the other category 0. Thus, a five‐category variable ranging from 0 to 4 was formed. For the interaction analyses, the variable was further categorised as follows: 0/1/2–4 unfavourable habits.

### Statistical analyses

**FIGURE 2:**
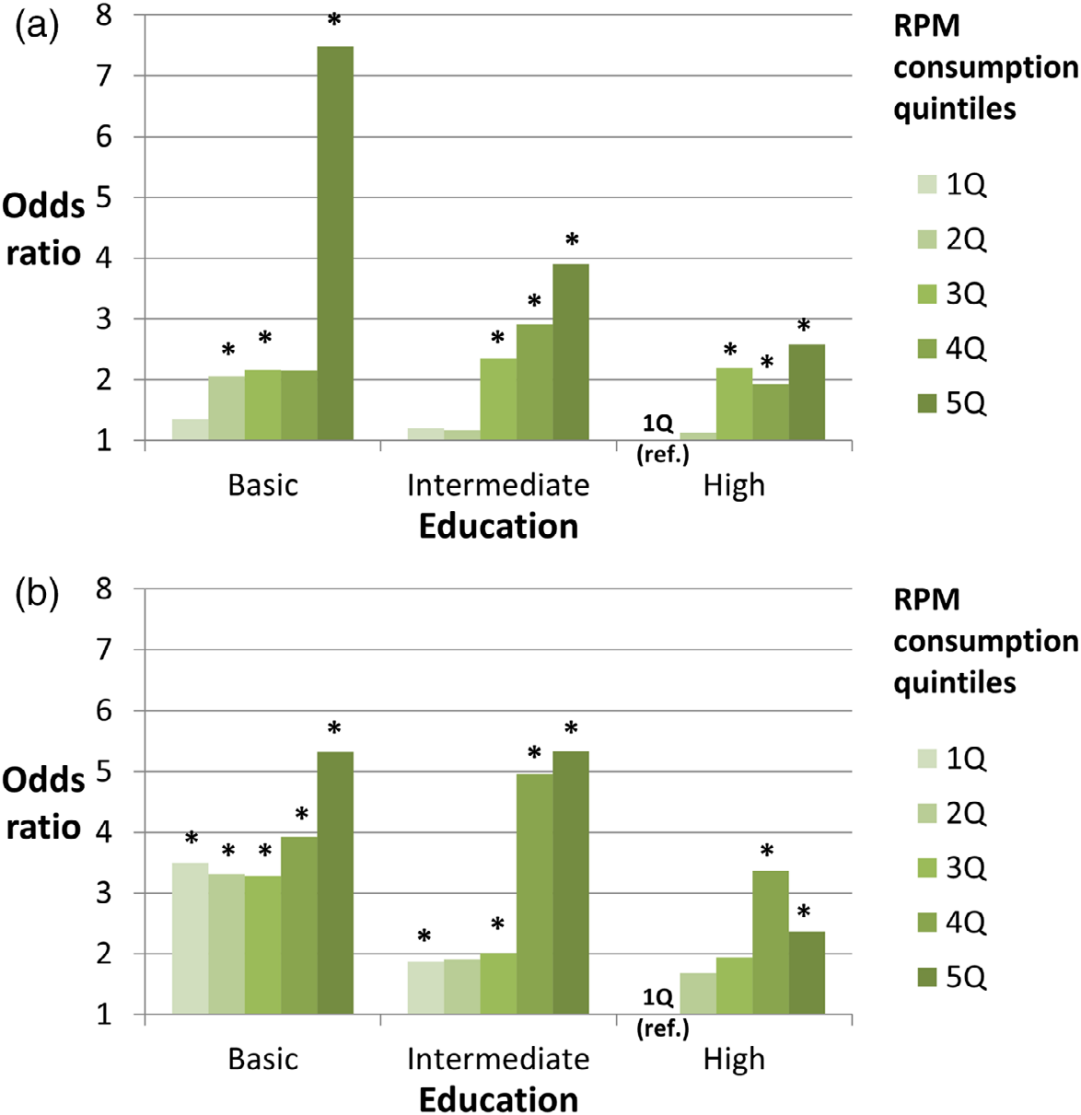
(a) Odds of obesity in education and red and processed meat (RPM) consumption groups in women (reference: lowest RPM consumption, high education). *Statistically significantly different from the reference (*p* < 0.05). Adjusted for age, energy intake, residential area, household income, household structure, employment status, leisure‐time physical activity (PA), vegetable, legume and fruit (VLF) consumption, alcohol consumption and smoking. (b) Odds of obesity in education and RPM consumption groups in men (reference: lowest RPM consumption, high education).

The linear regression model was used to study differences in population characteristics (Table 1) as well as in BMI and in RPM consumption (Table S1) between women and men and educational groups. The logistic regression model was used to study associations between RPM consumption and obesity in educational groups (Tables 2a and 2b), between interaction categories of RPM consumption and education, and obesity (Table 3, Figure 2a,b), between background variables and obesity (Table S2), between RPM consumption and obesity in household income quartiles (Table S3) and between RPM consumption and obesity in interaction categories of education and unfavourable lifestyle habits (Table S4).

**TABLE 1:** Characteristics of study population (*n* = 4494).

	Women					Men					
		Education					Education				
	All (*n* = 2480)	Basic (*n* = 360)	Inter‐mediate (*n* = 936)	High (*n* = 1184)	*p*	All (*n* = 2014)	Basic (*n* = 329)	Intermediate (*n* = 891)	High (*n* = 794)	*p*	*p* for sex‐difference
BMI (kg/m^2^), mean (SD)	26.8 (0.16)	28.2 (0.45)	27.0 (0.24)	26.1 (0.19)	**<0.001**	27.5 (0.21)	28.7 (0.35)	27.5 (0.37)	26.9 (0.18)	**<0.001**	**0.006**
Obesity (BMI ≥ 30 kg/m^2^), %	23.1	31.9	24.2	19.1	**<0.001**	25.1	35.3	25.4	19.5	**<0.001**	0.25
RPM consumption (g/day), mean (SD)	85.9 (2.03)	99.3 (9.75)	84.8 (2.70)	82.7 (2.26)	0.23	144.4 (2.33)	149.8 (7.02)	147.3 (3.53)	137.3 (3.03)	0.06	**<0.001**
Energy intake (kJ/day), mean (SD)	7771 (70.2)	7807 (230)	7552 (119)	7976 (76.1)	**0.004**	9713 (91.7)	9412 (238)	9781 (148)	9765 (121)	0.40	**<0.001**
Energy intake (kcal/day), mean (SD)	1857 (16.8)	1866 (55.0)	1805 (28.5)	1906 (18.2)	**0.004**	2321 (21.9)	2250 (56.8)	2338 (35.3)	2334 (28.9)	0.40	**<0.001**
Age (years), mean (SD)	46.8 (0.59)	56.4 (1.83)	44.4 (0.99)	46.0 (0.55)	**<0.001**	47.0 (0.73)	53.8 (1.87)	44.3 (1.05)	47.5 (0.79)	**<0.001**	0.86
Residential area, %											
Urban areas	62.8	58.3	57.4	69.7	**<0.001**	59.3	54.2	53.1	71.2	**<0.001**	0.14
Areas near urban areas, rural centres	22.4	21.9	24.3	20.6	0.32	25.9	26.3	29.2	20.6	**0.02**	0.11
Remote rural areas	14.8	19.8	18.4	9.66	**<0.001**	14.9	19.5	17.7	8.28	**0.006**	0.97
Household structure, %											
Living alone	24.6	35.1	25.1	20.7	**<0.001**	23.4	21.7	25.6	21.0	0.32	0.54
At least one adult and one child	30.1	17.1	27.9	36.5	**<0.001**	28.8	15.5	30.5	33.0	**<0.001**	0.46
Adults only	45.3	47.8	47.0	42.8	0.26	47.8	62.8	44.0	46.1	**<0.001**	0.20
Employment status, %											
Employed	52.7	23.8	46.2	68.6	**<0.001**	58.8	38.7	59.5	68.0	**<0.0001**	**0.003**
Other	47.3	76.2	53.8	31.4	**<0.001**	41.2	61.3	40.5	32.0	**<0.0001**	**0.003**
Household income quintiles, %											
1st (lowest)	23.4	38.2	30.6	12.1	**<0.001**	21.6	28.6	26.3	11.2	**<0.001**	0.40
2nd	16.1	16.9	17.9	14.2	0.09	21.4	27.3	24.7	13.8	**<0.001**	**<0.001**
3rd	21.6	25.1	24.9	17.3	**<0.001**	16.7	16.9	16.9	16.2	0.92	**<0.001**
4th	19.0	15.1	17.0	22.0	**<0.001**	18.8	18.0	18.8	19.0	0.95	0.91
5th (highest)	19.9	4.69	9.59	34.4	**<0.001**	21.6	9.14	13.2	39.8	**<0.001**	0.31
Leisure‐time PA, %											
Low	22.8	28.8	24.4	19.2	**0.006**	22.1	37.5	20.1	17.4	**<0.001**	0.69
Moderate or high	77.2	71.2	75.6	80.8	**0.006**	77.9	62.5	79.9	82.6	**<0.001**	0.69
VLF consumption quintiles, %											
1st (lowest)	20.8	25.2	24.0	16.3	**<0.001**	20.6	26.8	23.0	13.7	**<0.001**	0.88
2nd–5th	79.2	74.8	76.0	83.7	**<0.001**	79.4	73.2	77.0	86.3	**<0.001**	0.88
Alcohol consumption, %											
Risk use	18.1	15.8	20.1	16.9	0.33	33.2	29.0	35.9	31.2	0.08	**<0.001**
Moderate or low use	81.9	84.2	79.9	83.1	0.33	66.8	71.0	64.1	68.8	0.08	**<0.001**
Smoking, %											
Daily	12.4	19.6	15.2	7.26	**<0.001**	14.4	25.2	15.1	7.93	**<0.001**	0.10
Occasional or no	87.6	80.4	84.8	92.7	**<0.001**	85.6	74.8	84.9	92.1	**<0.001**	0.10
Unfavourable lifestyle habits^a^, %											
0	51.3	48.1	46.2	57.4	**<0.001**	42.1	33.7	38.7	51.3	**<0.001**	**<0.001**
1	30.6	27.0	32.1	30.4	0.31	34.8	30.7	37.2	33.3	0.18	**0.05**
2	12.6	16.7	15.1	8.70	**<0.001**	15.7	22.7	16.9	10.6	**<0.001**	**0.03**
3	4.11	5.97	5.00	2.63	**0.01**	5.72	8.24	5.89	4.27	0.22	0.08
4	1.38	2.27	1.61	0.86	0.14	1.66	4.67	1.35	0.68	**0.002**	0.53

*Note*: Bolded values are statistically significant.

Abbreviations: BMI, body mass index; PA, physical activity; RPM, red and processed meat; VLF, vegetable, legume and fruit.

^a^ Low leisure‐time PA, the lowest VLF consumption quintile, alcohol risk use, or daily smoking.

**TABLE 2a:** Odds ratio (OR) (95% confidence interval [CI]) of obesity (body mass index [BMI] ≥ 30 kg/m^2^) between red and processed meat (RPM) consumption quintiles in women in education categories.

			Education					
	All (*n* = 2476)		Basic (*n* = 359)		Intermediate (*n* = 935)		High (*n* = 1182)	
	*N*/*n*	OR (95% CI)	*N*/*n*	OR (95% CI)	*N*/*n*	OR (95% CI)	*N*/*n*	OR (95% CI)
Adjustment Model 1^a^								
RPM quintiles								
1st (lowest), reference	85/494	1	16/67	1	32/159	1	37/268	1
2nd	97/496	1.09 (0.79–1.50)	21/82	1.07 (0.46–2.50)	36/179	0.90 (0.51–1.58)	40/235	1.20 (0.72–2.01)
3rd	129/496	**1.85 (1.31–2.62)**	21/64	1.47 (0.65–3.31)	53/188	**1.89 (1.08–3.30)**	55/244	**1.86 (1.12–3.09)**
4th	134/495	**2.04 (1.44–2.87)**	21/67	1.66 (0.72–3.86)	57/183	**2.03 (1.19–3.45)**	56/245	**2.07 (1.16–3.69)**
5th (highest)	167/495	**3.37 (2.38–4.76)**	33/79	**2.93 (1.29–6.65)**	82/226	**2.97 (1.74–5.08)**	52/190	**2.89 (1.73–4.85)**
*p* for heterogeneity		**<0.001**		**0.03**		**<0.001**		**<0.001**
*p* for education interaction								0.93
Adjustment Model 2^b^								
RPM quintiles								
1st (lowest), reference	82/481	1	14/63	1	31/154	1	37/264	1
2nd	90/471	1.08 (0.76–1.53)	18/68	1.16 (0.46–2.95)	35/174	0.90 (0.50–1.60)	37/229	1.16 (0.68–1.97)
3rd	125/486	**1.83 (1.27–2.66)**	21/62	1.71 (0.68–4.34)	49/182	1.71 (0.93–3.14)	55/242	**2.02 (1.21–3.38)**
4th	130/481	**2.07 (1.47–2.93)**	20/64	1.55 (0.65–3.67)	55/177	**2.04 (1.19–3.49)**	55/240	**2.18 (1.18–4.03)**
5th (highest)	157/466	**3.34 (2.32–4.82)**	31/71	**3.52 (1.38–9.00)**	76/210	**2.96 (1.67–5.23)**	50/185	**2.91 (1.67–5.07)**
*p* for heterogeneity		**<0.001**		**0.04**		**<0.001**		**<0.001**
*p* for education interaction								0.88
Adjustment Model 3^c^								
RPM quintiles								
1st (lowest), reference	79/469	1	14/62	1	29/149	1	36/258	1
2nd	88/462	1.13 (0.78–1.65)	18/66	1.34 (0.53–3.40)	34/172	0.95 (0.51–1.76)	36/224	1.11 (0.63–1.96)
3rd	124/475	**2.01 (1.36–2.97)**	20/61	1.56 (0.54–4.46)	49/179	1.94 (0.99–3.77)	55/235	**2.25 (1.34–3.77)**
4th	128/475	**2.08 (1.44–3.03)**	20/64	1.38 (0.56–3.42)	53/173	**2.27 (1.22–4.23)**	55/238	**1.95 (1.03–3.69)**
5th (highest)	154/462	**3.39 (2.27–5.05)**	31/71	**3.97 (1.49–10.5)**	75/209	**2.97 (1.65–5.33)**	48/182	**2.66 (1.50–4.73)**
*p* for heterogeneity		**<0.001**		**0.04**		**<0.001**		**<0.001**
*p* for education interaction								0.82

*Note*: Bolded values are statistically significant. *n*, individuals in the category; *N*, obesity cases in the category.

^a^ Adjusted for age and energy intake.

^b^ Adjusted for age, energy intake, residential area, household income, household structure and employment status.

^c^ Adjusted for age, energy intake, residential area, household income, household structure, employment status, leisure‐time physical activity (PA), vegetable, legume and fruit (VLF) consumption, alcohol consumption and smoking.

**TABLE 2b:** Odds of obesity (body mass index [BMI] ≥ 30 kg/m^2^) between red and processed meat (RPM) consumption quintiles in men in education categories.

			Education					
	All (*n* = 2005)		Basic (*n* = 329)		Intermediate (*n* = 884)		High (*n* = 792)	
	*N*/*n*	OR (95% CI)	*N*/*n*	OR (95% CI)	*N*/*n*	OR (95% CI)	*N*/*n*	OR (95% CI)
Adjustment Model 1^a^								
RPM quintiles								
1st (lowest), reference	72/398	1	15/57	1	32/156	1	25/185	1
2nd	90/402	1.28 (0.85–1.94)	21/66	1.27 (0.53–3.07)	38/177	1.00 (0.54–1.84)	31/159	1.58 (0.85–2.96)
3rd	92/402	1.26 (0.81–1.95)	18/59	1.07 (0.43–2.66)	41/185	0.98 (0.52–1.87)	33/158	**1.85 (1.02–3.37)**
4th	127/403	**2.62 (1.68–4.08)**	23/66	1.39 (0.56–3.46)	54/169	**2.62 (1.37–5.03)**	50/168	**3.23 (1.83–5.69)**
5th (highest)	147/400	**2.87 (1.94–4.25)**	41/81	2.42 (0.94–6.24)	75/197	**2.68 (1.56–4.60)**	31/122	**2.30 (1.23–4.32)**
*p* for heterogeneity		**<0.001**		0.21		**<0.001**		**0.001**
*p* for education interaction								0.51
Adjustment Model 2^b^								
RPM quintiles								
1st (lowest), reference	70/385	1	15/51	1	31/151	1	24/183	1
2nd	88/396	1.22 (0.79–1.88)	20/63	0.99 (0.40–2.44)	37/174	1.01 (0.54–1.89)	31/159	1.73 (0.91–3.27)
3rd	89/392	1.24 (0.78–1.95)	16/52	1.08 (0.41–2.85)	40/183	0.99 (0.51–1.94)	33/157	**1.95 (1.04–3.63)**
4th	126/399	**2.51 (1.63–3.86)**	22/65	1.17 (0.45–3.01)	54/167	**2.66 (1.37–5.14)**	50/167	**3.39 (1.92–5.97)**
5th (highest)	141/385	**2.68 (1.81–3.98)**	38/77	2.13 (0.79–5.77)	73/191	**2.85 (1.67–4.86)**	30/117	**2.46 (1.29–4.71)**
*p* for heterogeneity		**<0.001**		0.28		**<0.001**		**<0.001**
*p* for education interaction								0.38
Adjustment Model 3^c^								
RPM quintiles								
1st (lowest), reference	69/380	1	15/49	1	31/150	1	23/181	1
2nd	88/394	1.22 (0.78–1.90)	20/63	0.90 (0.37–2.19)	37/172	1.00 (0.53–1.88)	31/159	1.84 (0.94–3.57)
3rd	89/387	1.29 (0.80–2.07)	16/51	0.92 (0.31–2.74)	40/181	1.06 (0.54–2.07)	33/155	**2.13 (1.09–4.17)**
4th	126/398	**2.49 (1.61–3.85)**	22/65	1.01 (0.39–2.66)	54/167	**2.57 (1.32–5.01)**	50/166	**3.69 (2.02–6.73)**
5th (highest)	138/379	**2.63 (1.77–3.90)**	36/75	1.59 (0.59–4.28)	72/187	**2.88 (1.65–5.00)**	30/117	**2.59 (1.31–5.13)**
*p* for heterogeneity		**<0.001**		0.58		**<0.001**		**<0.001**
*p* for education interaction								0.35

*Note*: Bolded values are statistically significant. *n*, individuals in the category; *N*, obesity cases in the category.

Abbreviations: CI, confidence interval; OR, odds ratio.

^a^ Adjusted for age and energy intake.

^b^ Adjusted for age, energy intake, residential area, household income, household structure and employment status.

^c^ Adjusted for age, energy intake, residential area, household income, household structure, employment status, leisure‐time physical activity (PA), vegetable, legume and fruit (VLF) consumption, alcohol consumption and smoking.

**TABLE 3:** Odds of obesity (body mass index [BMI] ≥ 30 kg/m^2^) between interaction categories of educational groups and red and processed meat (RPM) consumption quintiles in women and in men.^a^

	Education		
	Basic (OR (95% CI))	Intermediate (OR (95% CI))	High (OR (95% CI))
*Women*			
RPM quintiles			
1st (lowest), reference	1.34 (0.63–2.85)	1.20 (0.65–2.22)	1 (reference)
2nd	**2.06 (1.10–3.87)**	1.17 (0.65–2.12)	1.13 (0.65–1.96)
3rd	**2.16 (1.05–4.45)**	**2.35 (1.28–4.34)**	**2.20 (1.32–3.67)**
4th	2.15 (0.89–5.21)	**2.91 (1.59–5.32)**	**1.93 (1.07–3.48)**
5th	**7.48 (2.74–20.4)**	**3.90 (2.18–6.96)**	**2.59 (1.51–4.42)**
*Men*			
RPM quintiles			
1st (lowest), reference	**3.49 (1.49–8.22)**	**1.88 (1.01–3.47)**	1
2nd	**3.32 (1.49–7.38)**	1.90 (0.99–3.67)	1.68 (0.88–3.22)
3rd	**3.27 (1.39–7.70)**	**2.01 (1.04–3.91)**	1.94 (0.99–3.82)
4th	**3.93 (1.92–8.04)**	**4.96 (2.58–9.55)**	**3.37 (1.81–6.26)**
5th	**5.32 (2.54–11.1)**	**5.33 (2.85–9.99)**	**2.36 (1.21–4.58)**

*Note*: Bolded values are statistically significant.

Abbreviations: CI, confidence interval; OR, odds ratio.

^a^ Adjusted for age, energy intake, residential area, household income, household structure, employment status, leisure‐time physical activity (PA), vegetable, legume and fruit (VLF) consumption, alcohol consumption and smoking.

Main analyses in this study were conducted using a logistic regression model, categorised variables and, in the case of dietary variables, using energy‐adjusted values [46]. Linear and logistic regression models are commonly used methods to study relationships between dependent and independent variables. While linear regression is used to study relationships where dependent variable is continuous, logistic regression is used to predict the probability of a binary outcome. In this study, the large sample size and independent observations enabled the use of these models.

For the interaction analyses, a 15‐class interaction variable containing education variable and RPM quintile variable was formed (Table 3, Figure 2a,b). The strength of association was estimated with means or prevalences and odds ratios (OR). The effect modification of education in the association between RPM consumption and obesity, was studied by including in the model an interaction term between the education and the RPM quintile variable. Similarly, the effect modification of the lifestyle composite variable and income in the association between RPM consumption and obesity was studied by including in the model an interaction term between the potential effect modifier in question and RPM variable.

In the logistic models, three adjustment models were used. In Model 1, age in 10‐year categories and energy intake (kJ) as a continuous variable were adjusted for. In Model 2, residential area, household structure, employment status and household income were added to the model. In Model 3, binary unfavourable lifestyle variables (low leisure‐time PA, low VLF consumption, alcohol risk use and daily smoking) were additionally included. Energy intake was included in Model 1 to control for the association between energy intake and obesity. All variables were included in the final model to reveal the association between RPM consumption and obesity independent of other lifestyle factors.

All analyses were conducted separately for men and women due to significant differences in RPM consumption, except for an interaction analysis for education and accumulation of unfavourable lifestyle habits, in which the number of participants restricted separate analyses. Analysis weights were used to mitigate non‐participation bias [49].

All of the analyses were conducted with SAS Enterprise Guide, version 7.15 HF7 (SAS Institute Inc.).

### Ethical considerations

The FinHealth 2017 Study was performed in accordance with the Declaration of Helsinki and approved by the Ethics Committee of Helsinki and Uusimaa Hospital District. All participants gave their written informed consent.

## RESULTS

On average, women had lower BMI (26.8 kg/m^2^) than men (27.5 kg/m^2^) (*p* for difference = 0.006) (Table 1). As expected, BMI also showed a notable inverse gradient across education groups, with BMI being approximately two units higher in women and men with basic education compared to women and men with high education (*p* for difference <0.0001) (Table 1). RPM consumption was also lower in women (86 g/day) than in men (144 g/day) (*p* for difference <0.0001), but no statistically significant differences emerged between education groups (Table 1). Most of the unfavourable lifestyle habits were more common among individuals with basic education than in individuals with high education, except for alcohol risk use, which showed no differences between education groups (Table 1).

In men and women, BMI was higher in the age group of 55–74‐year olds, in those living in rural areas and in those with high RPM consumption than in individuals in other categories of the variables in question (Table S1). Additionally, in women, BMI was higher in those living in households with adults only and those who were not employed. Of the lifestyle habit variables, only leisure‐time PA showed BMI differences, with BMI being higher in women and in men with low PA. Of the accumulation categories of unfavourable lifestyle habits, BMI was lowest in women with no such habits. In men, no statistically significant differences appeared between accumulation categories.

RPM consumption was higher in 35–64‐year olds, those living in remote rural areas, those with underage children in the household, those with low VLF consumption, daily smokers and individuals with obesity than in individuals in respective population categories in men and women (Table S1). RPM consumption was lowest in men and women with no unfavourable lifestyle habits and grew fairly linearly through accumulation categories.

Supplementary analyses testing odds of obesity between categories of each background variable in a complete model proved that after adjusting for other factors, odds of obesity remained at a statistically significantly elevated level in women and in men in the oldest age group, with basic education or with low leisure‐time PA compared to those in the corresponding reference groups (Table S2). Moreover, daily smoking showed an inverse association with obesity. In men only, those living in areas near urban areas or in rural centres had higher odds of obesity than men living in urban areas. In women only, those living in households with at least one underage child had lower odds of obesity compared to women living alone.

Odds of obesity grew in parallel with RPM consumption quintiles in women (OR (95% confidence interval [CI]) for the highest vs. the lowest quintile: 3.37 (2.38–4.76)) and men (OR (95% CI) for the highest vs. the lowest quintile: 2.87 (1.94–4.25)) with adjustment for age and energy intake (Model 1) (Tables 2a and 2b). A model where only age was adjusted for was tested, but the results did not differ from a model where energy intake was included. Additionally, Model 2 without income was tested, with no notable changes to the results. When adjusting for socio‐economic and ‐demographic variables (Model 2) and additionally for lifestyle factors (Model 3), the results appeared rather similar. In the analyses stratified by education, the results in women and in men in each education category were directed towards the same direction as in the whole population, with higher odds of obesity in the highest RPM quintile. Analyses with household income in three categories as a stratifying variable (Table S3) showed fairly similar results as the corresponding primary analyses using education as a stratifying variable.

In further analysis comparing the odds of obesity simultaneously between categories of RPM consumption and education to the reference category of lowest RPM consumption and high education, the odds appeared notably higher in those with basic education and the highest RPM consumption (OR (95% CI) among women: 7.48 (2.74–20.4) and among men: 5.32 (2.54–11.1)) (Table 3, Figure 2a,b). However, only 3.2% of women and 4.0% of men belonged to the group with basic education and the highest RPM consumption. Interactions between RPM quintiles and education as well as between RPM quintiles, education and sex were tested, and they appeared non‐significant as within each educational group, odds of obesity grew along RPM quintiles (Tables 2a and 2b).

Interaction analyses among RPM consumption tertiles, education and lifestyle composite variable (in three categories) showed no statistically significant interactions (Table S4). In the high and intermediate education groups, odds of obesity were elevated within the highest RPM tertile compared to the lowest tertile, regardless of the number of unfavourable lifestyle habits. An exception was the group with high education and 2–4 unfavourable habits, which did not differ statistically significantly from the reference group but in which a tendency towards elevated odds of obesity could also be seen. Within the basic education group, none of the comparisons were statistically significant.

## DISCUSSION

In this article, we examined how the association between RPM consumption and obesity varied across educational groups and how accumulation of other unfavourable lifestyle habits modified the association. The novelty of this study was the simultaneous examination of these factors. Our main findings showed that higher RPM consumption was independently associated with a higher risk of obesity in women and men, regardless of educational level or other unfavourable lifestyle habits. Moreover, compared to individuals with high education and low RPM consumption, it appeared that individuals with basic education and high RPM consumption had five‐ to sevenfold odds of obesity.

The results on the direct association between RPM consumption and odds of obesity were in line with previous literature [5, 6, 50]. In their systematic review and meta‐analysis, including 113,477 individuals from 18 international studies, Rouhani et al. [6] concluded that higher consumption of RPM was significantly associated with a higher risk of obesity and higher BMI and waist circumference. In general, overconsumption of foods, which leads to excess energy intake and obesity, also consumes natural resources and causes unnecessary climate impact [51]. RPM products have fairly high energy density. In addition, a Western/unhealthy dietary style, in which RPM consumption is typically high, also includes other foods that contribute to obesity, such as sugar‐sweetened beverages and refined grains [52], and less healthy foods such as fruits and vegetables [50]. Moreover, a Western dietary style has been associated with other lifestyle habits contributing to obesity, such as lower PA [52]. We also found that those in the highest RPM consumption quintile were more likely to accumulate other unhealthy habits.

In many studies, SES has shown an inverse gradient with obesity and many unhealthy lifestyle habits [30, 31]. Our results showed that within each educational group, the odds of obesity increased along with higher RPM consumption, and this finding remained even when other lifestyle habits were accounted for. Only in men with basic education the differences did not reach statistical significance. In men in this study, a generally higher level of RPM consumption and obesity presumably results in more moderate differences between groups. Sex differences exist in lifestyle habits, including RPM consumption, and women's food preferences as consumers appear to be more sustainable than men's [39]. The ideals of masculinity, strength and power have traditionally been associated with meat consumption [38, 53–55], which, in addition to generally greater food consumption, is presumably largely responsible for greater RPM consumption in men.

Individual level, community level, socio‐economic and sociocultural contributors interact in an individual's susceptibility to obesity [56]. Low education may contribute to greater odds of obesity through poorer health literacy [57], poorer lifestyle habits [31] and also indifference towards obtaining a healthy lifestyle that would prevent obesity. In addition, RPM consumption among those with basic education may consist of different types of meat and more processed and energy‐dense meat compared to those with high education [58]. Lallukka et al. [30] concluded that healthy dietary habits were less common in individuals with low SES than in respective reference groups. In another Finnish study, Konttinen et al. [33] examined the motives behind food selections and found that among individuals with higher academic education, the motives were more based on health and ethical concerns, whereas individuals with lower education valued more familiarity and lower price. Prices of foods do matter, and results of an umbrella review study concluded that higher taxation on unhealthy foods could reduce socio‐economic inequalities in diets and thus in population health [59].

Even though having multiple unfavourable lifestyle habits was relatively rare in our study population, their accumulation was more frequent in those with basic education. This is in line with previous findings [26, 27, 28, 29]. Another Finnish study drew aligned conclusions with those with up to 9 years of education having over twofold odds of having 3–4 unfavourable habits compared to those with over 13 years of education [60]. Further, a recent systematic review including 26 international studies concluded that people with low SES are more likely to have multiple unfavourable lifestyle habits, and that such behaviours are associated with higher allostatic load, resulting in higher risk of several chronic diseases and premature mortality [61]. Even though previous studies have indicated that multiple unfavourable lifestyle habits contribute to an increased risk of morbidity, adjusting for such habits did not attenuate the associations between RPM consumption and obesity at all, possibly due to different associations between each unfavourable habit and obesity. Previous studies have shown a positive association between PA and weight maintenance [62], ambiguous associations between alcohol consumption [63] or smoking [64] and obesity and an inverse association between fruit and vegetable consumption and obesity [5]. VLF consumption is prone to overreporting, especially among individuals with obesity [65], which could explain the insignificance between VLF consumption and obesity found in this study.

As the inclusion of lifestyle factors in the model did not attenuate the association in most of the groups in this study, it can be interpreted that, generally, the accumulation of other such unfavourable lifestyle habits does not explain the positive association between RPM consumption and obesity. An exception was men with basic education, among whom, supposedly, the odds of obesity are already elevated; other unfavourable lifestyle habits contribute to it, and high RPM consumption does not play that meaningful a role as in other educational groups or in women. Men with basic education had more unhealthy lifestyle habits in this study and generally in previous studies [26, 27, 28, 29], and thus the impact of other lifestyles may be relatively greater than in other groups, even though RPM consumption also contributes to the risk.

Analyses of this study showed an inverse association between education and obesity, even after adjusting for confounding factors (including income). In corresponding analyses (including adjusting for education), income was not related to obesity among women, whereas in men, only two middle quintiles showed lower odds of obesity. These results were in line with previous studies indicating that low education is a more coherent predictor of obesity than low income is [66]. Different mechanisms, including health literacy or values, may explain such differences between different SES indicators. A Finnish study indicated that barriers to consuming plant‐based diets are the most prevalent in those with low education [67]. Interestingly, another Finnish study found that those with the highest yearly incomes were less interested in the environmental impacts of their diets (over €40,000/year) or less interested in favouring plant‐based diets (over €30,000/year) compared to those with lower yearly incomes [39]. Findings of these studies also suggest that differences exist between different SES variables in readiness to change one's diet in a more sustainable direction. Despite these differences, supplementary stratified analyses by income, conducted to study the association from other SES aspects (Table S3), showed the odds of obesity elevating in parallel with RPM consumption in each income category also after adjusting for confounding factors in most of the groups.

The findings of this study agreed with the broader context of existing research on associations between unecological and unhealthy diet and obesity [68]. Even though such unsustainable diets may contribute to obesity, obesity can also be seen as unsustainable, as unnecessary excess energy intake that leads to obesity (Metabolic Food Waste) has impact on the environment [51]. In line with the literature [5, 6, 50], this study found an association between a higher consumption of RPM and a greater risk of obesity. In addition, findings of this study showed that this association remained significant regardless of level of education or unfavourable health behaviours. Even though SES has shown an inverse gradient with obesity [20, 21, 22] and many unhealthy lifestyle habits in previous studies [30, 31], it appears that the direct association between consumption of RPM and obesity is independent of these other factors, and decreasing the consumption of RPM could benefit population health and reduce climate impact in each SES group and regardless of existence of other health behaviours.

This study involved certain strengths. We utilised data from a large health examination study based on a nationally representative adult population sample and included a comprehensive set of information on participants [40]. Measured information on weight and height prevented the possibility of error caused by self‐reporting [69]. Validated and updated FFQ produced information on RPM consumption [41, 42, 43]. Generally, the value of this study to the literature is the simultaneous scrutiny of RPM consumption, obesity, education, and the accumulation of certain other unfavourable lifestyle factors. It is of importance to take these factors simultaneously into account to be able to implement population health‐ and sustainability‐promoting policies in different population groups.

Some methodological issues, however, should also be considered. First, we do not provide any causal evidence on the associations. Second, there might be some lifestyle factors or eating habits that are not observed in our data. Third, as this study employed cross‐sectional study design, future studies should investigate time trends in healthy and ecological eating patterns across SES groups. Fourth, despite relatively good participation rate in the FinHealth 2017 Study (58% participated in the health examination), non‐participation – commonly occurring in population surveys – may have biased the results towards more conservative. In this study, however, analysis weights were used to mitigate this problem [49]. Fifth, the number of individuals in some of the interaction groups is low, and thus those results should be interpreted with caution. Sixth, the FFQ method may cause recall bias, misestimate absolute levels of food and energy intake and, thus, attenuate associations in nutritional epidemiology studies [70]. However, FFQ is a suitable tool to order individuals according to their intake and compare such groups [46], which was the aim in this study. To mitigate possible bias related to the FFQ method, the FFQ – originally designed in 1996 – has been validated against food records and updated regularly [41, 42, 43]. Seventh, dietary misreporting, consisting of under‐ and overreporting, may have affected the results; individuals tend to underreport foods considered unhealthy and overreport foods considered healthy, and the misreporting appears more prevalent among individuals with obesity [65, 71]. Using Willett's residual method [72] for RPM consumption and VLF consumption variables and adjusting for energy intake in the analyses mitigated the effect of potential energy misreporting. Eighth, due to rather similar environmental impacts, it was decided that the RPM consumption variable would comprise all RPM in this study, which is also consistent with previous studies, and appears to be an adequate indicator of consumption [73, 74]. As different RPM products, however, may have divergent associations with obesity [73], and sources of RPM may differ according to educational groups, further studies should address the questions examined in this study using different types of RPM as exposures. Ninth, low VLF consumption was chosen to represent poor quality of diet. Even though it is a crude proxy indicator of the whole diet omitting, for example intake of refined grains and sugar‐sweetened beverages, consumption of VLF has been shown to associate with a reduced risk of adiposity [5]. Tenth, three adjustment models with carefully selected confounders were used in the analyses. Despite this, the possibility of residual confounding exists. For instance, more comprehensive adjustment for dietary factors (e.g. consumption of sugary products) or other lifestyle factors (e.g. sleep or work‐related PA) could have affected the results.

## CONCLUSION

It seems that RPM consumption is a strong predictor of obesity regardless of educational attainment or other lifestyle factors, suggesting that individuals in each educational group could benefit from reducing RPM consumption. The combination of low education and high RPM consumption, however, seems to relate to even higher odds of obesity. Thus, even though causality between these factors cannot be proved based on an observational study such as this, these results add to the literature indicating that, in addition to promoting healthy and climate‐wise dietary change, investing in educational system policy is a matter of great importance in terms of preventing obesity and improving population health. A decrease in RPM consumption could benefit both population health and mitigating climate change, and this double benefit should be emphasised in promoting dietary transition as well as in health policy. Lehikoinen and Salonen [39] studied food preferences among Finns and found that although environmental values exclusively scored lower, when health motives were combined with environmental motives, together they scored higher. The results suggest that underlining combined environment and health benefits could be more effective in sustainable dietary transition strategies targeted at consumers. In order to succeed in reaching and impacting groups with low education or low income, customised public health programmes recognising the special characteristics and needs of such groups should be tailored. Accounting for structural barriers between groups includes providing comprehensible and pragmatic information on healthy and sustainable health behaviours and economically increasing opportunities to purchase healthy food through policy measures and taxation.

## AUTHOR CONTRIBUTIONS

*Conceptualisation; data curation; formal analysis; investigation; methodology; project administration; visualisation; writing – original draft; writing – review and editing*: Laura Sares‐Jäske. *Conceptualisation; data curation; formal analysis; methodology; software; writing – review and editing*: Heli Tapanainen. *Conceptualisation; funding acquisition; resources; supervision; writing – review and editing*: Liisa Valsta. *Investigation; writing – original draft; writing – review and editing*: Peppi Haario. *Funding acquisition; writing – review and editing*: Satu Männistö. *Conceptualisation; funding acquisition; investigation; methodology; supervision; writing – original draft; writing – review and editing*: Maria Vaalavuo.

## CONFLICT OF INTEREST STATEMENT

The authors declare no conflicts of interest.

## ETHICS STATEMENT

The FinHealth 2017 Study was performed in accordance with the Declaration of Helsinki and approved by the Ethics Committee of Helsinki and Uusimaa Hospital District. All participants gave their written informed consent.

## Supporting information

Table S1 BMI and red and processed meat consumption in categories of selected sociodemographic, socio‐economic and lifestyle habits in women and men (*n* = 4494).

Table S2 Odds of obesity (BMI ≥ 30 kg/m^2^) between categories of background variables in women and men in a multivariate model^a^.

Table S3 OR (95% CI) of obesity (BMI ≥ 30 kg/m^2^) between red and processed meat consumption quintiles in women and men in household income quartiles.

Table S4 Odds of obesity (BMI ≥ 30 kg/m^2^) between red and processed meat consumption tertiles in interaction categories of education and unfavourable lifestyle habits (*n* = 4281).

## Data Availability

The data that support the findings of this study are available from the Finnish Social and Health Data Permit Authority Findata. Restrictions apply to the availability of these data, which were used under license for this study. Data are available at https://findata.fi/en/ with the permission of Findata.
