# Supplementary material for: Meat consumption and obesity: A climate‐friendly way to reduce health inequalities
Source: Public Health Chall. 2024 Mar 15;3(1):e163. doi: 10.1002/puh2.163 (PMC12060756; doi:10.1002/puh2.163)
Supplement: Supplementary file 1 — Table S1 BMI and red and processed meat consumption in categories of selected sociodemographic, socio‐economic and lifestyle habits in women and men (n = 4494). [file PUH2-3-e163-s003.docx]

| **Table S1.** BMI and red and processed meat consumption in categories of selected sociodemographic and –economic and lifestyle habits in men and in women (n=4494). | | | | | | | | | | |
| --- | --- | --- | --- | --- | --- | --- | --- | --- | --- | --- |
|  | **BMI (kg/m^2^)** | | | | |  | **RPM (g/day)** | | |  |
|  | **Women**  **n=2480** | |  | **Men**  **n=2014** | |  | **Women**  **n=2480** |  | **Men**  **n=2014** |  |
|  | **n** | **Mean (SE)** | | **n** | **Mean (SE)** | | **Mean (SE)** | | **Mean (SE)** | |
| Age (years) | 2480 |  | | 2014 |  | |  | |  | |
| 18-34 | 400 | 24.9 (0.35) | | 297 | 26.3 (0.70) | | 84.6 (6.08) | | 137.1 (6.21) | |
| 35-54 | 913 | 27.0 (0.19) | | 708 | 27.7 (0.17) | | 91.4 (2.27) | | 152.6 (3.78) | |
| 55-74 | 1167 | 28.1 (0.17) | | 1009 | 28.2 (0.13) | | 81.2 (1.67) | | 141.4 (3.33) | |
| P for heterogeneity |  | **<0.001** | |  | **0.004** | | **<0.001** | | **0.04** | |
| P for sex-interaction |  |  | |  | 0.06 | |  | | 0.65 | |
|  |  |  | |  |  | |  | |  | |
| Residential area | 2480 |  | | 2013 |  | |  | |  | |
| Urban areas | 1486 | 26.3 (0.18) | | 1162 | 27.0 (0.14) | | 80.2 (2.98) | | 138.4 (3.11) | |
| Areas near urban areas, rural centres | 573 | 27.3 (0.27) | | 500 | 28.4 (0.69) | | 89.0 (3.34) | | 149.3 (3.75) | |
| Remote rural areas | 421 | 28.1 (0.47) | | 351 | 28.0 (0.31) | | 105.7 (3.82) | | 160.1 (6.57) | |
| P for heterogeneity |  | **<0.001** | |  | **0.002** | | **<0.001** | | **0.004** | |
| P for sex-interaction |  |  | |  | 0.38 | |  | | 0.77 | |
|  |  |  | |  |  | |  | |  | |
| Household structure | 2470 |  | | 2008 |  | |  | |  | |
| Living alone | 584 | 26.6 (0.37) | | 399 | 27.2 (0.29) | | 71.8 (3.43) | | 139.4 (5.99) | |
| At least one adult and one child | 676 | 26.1 (0.20) | | 524 | 27.7 (0.54) | | 97.2 (5.19) | | 153.5 (3.89) | |
| Adults only | 1210 | 27.3 (0.22) | | 1085 | 27.5 (0.19) | | 86.0 (2.20) | | 141.3 (3.13) | |
| P for heterogeneity |  | **<0.001** | |  | 0.66 | | **<0.001** | | **0.02** | |
| P for sex-interaction |  |  | |  | **0.04** | |  | | 0.35 | |
|  |  |  | |  |  | |  | |  | |
| Employment status | 2473 |  | | 2012 |  | |  | |  | |
| Employed | 1347 | 26.5 (0.16) | | 1156 | 27.3 (0.17) | | 87.7 (1.73) | | 152.5 (3.11) | |
| Other | 1126 | 27.1 (0.27) | | 856 | 27.8 (0.44) | | 84.1 (3.80) | | 132.8 (3.79) | |
| P for heterogeneity |  | **0.03** | |  | 0.30 | | 0.38 | | **<0.001** | |
| P for sex-interaction |  |  | |  | 0.73 | |  | | **0.01** | |
|  |  |  | |  |  | |  | |  | |
| Household income | 2392 |  | | 1968 |  | |  | |  | |
| 1st (lowest) | 412 | 26.4 (0.45) | | 315 | 28.2 (0.81) | | 88.4 (5.10) | | 143.3 (7.52) | |
| 2nd | 387 | 27.4 (0.37) | | 393 | 26.9 (0.32) | | 94.0 (3.24) | | 149.9 (4.90) | |
| 3rd | 587 | 27.5 (0.27) | | 382 | 27.5 (0.22) | | 80.7 (2.11) | | 142.4 (4.50) | |
| 4th | 496 | 26.6 (0.26) | | 399 | 27.8 (0.26) | | 85.1 (2.84) | | 148.4 (4.62) | |
| 5th (highest) | 510 | 26.0 (0.25) | | 479 | 27.1 (0.27) | | 75.2 (3.36) | | 139.5 (4.06) | |
| P for heterogeneity |  | **0.001** | |  | 0.25 | | **<0.001** | | 0.41 | |
| P for sex-interaction |  |  | |  | **0.009** | |  | | 0.77 | |
|  |  |  | |  |  | |  | |  | |
| Leisure-time PA | 2452 |  | | 2005 |  | |  | |  | |
| Low | 568 | 28.8 (0.35) | | 429 | 28.8 (0.34) | | 95.5 (7.33) | | 149.7 (6.32) | |
| Moderate or high | 1884 | 26.2 (0.17) | | 1576 | 27.1 (0.25) | | 83.3 (2.08) | | 143.0 (2.53) | |
| P for heterogeneity |  | **<0.001** | |  | **<0.001** | | 0.13 | | 0.34 | |
| P for sex-interaction |  |  | |  | 0.08 | |  | | 0.62 | |
|  |  |  | |  |  | |  | |  | |
| VLF consumption quintiles | 2480 |  | | 2014 |  | |  | |  | |
| 1st (lowest) | 496 | 27.1 (0.33) | | 402 | 27.4 (0.32) | | 99.5 (7.04) | | 157.3 (6.89) | |
| 2nd – 5th | 1984 | 26.7 (0.19) | | 1612 | 27.5 (0.26) | | 82.4 (2.09) | | 141.1 (2.40) | |
| P for heterogeneity |  | 0.39 | |  | 0.72 | | **0.03** | | **0.03** | |
| P for sex-interaction |  |  | |  | 0.39 | |  | | 0.94 | |
|  |  |  | |  |  | |  | |  | |
| Alcohol consumption | 2465 |  | | 2008 |  | |  | |  | |
| Risk use | 402 | 27.0 (0.39) | | 616 | 27.6 (0.28) | | 93.1 (4.60) | | 148.2 (4.25) | |
| Moderate or low use | 2063 | 26.7 (0.16) | | 1392 | 27.4 (0.27) | | 84.5 (2.14) | | 142.6 (2.94) | |
| P for heterogeneity |  | 0.51 | |  | 0.55 | | 0.07 | | 0.29 | |
| P for sex-interaction |  |  | |  | 0.92 | |  | | 0.67 | |
|  |  |  | |  |  | |  | |  | |
| Smoking | 2465 |  | | 1998 |  | |  | |  | |
| Daily | 286 | 26.9 (0.43) | | 294 | 27.5 (0.31) | | 93.7 (4.65) | | 167.6 (7.65) | |
| Occasional or no | 2179 | 26.8 (0.17) | | 1704 | 27.5 (0.24) | | 83.7 (1.94) | | 140.5 (2.19) | |
| P for heterogeneity |  | 0.82 | |  | 0.96 | | **0.05** | | **<0.001** | |
| P for sex-interaction |  |  | |  | 0.90 | |  | | 0.07 | |
|  |  |  | |  |  | |  | |  | |
| Unfavorable lifestyle habits^a^ | 2424 |  | | 1983 |  | |  | |  | |
| 0 | 1269 | 26.2 (0.22) | | 875 | 27.0 (0.43) | | 79.6 (2.43) | | 137.9 (2.53) | |
| 1 | 748 | 27.0 (0.30) | | 672 | 27.7 (0.26) | | 88.1 (3.40) | | 144.1 (4.45) | |
| 2 | 284 | 28.0 (0.51) | | 299 | 28.2 (0.37) | | 95.2 (4.37) | | 148.5 (6.10) | |
| 3 | 94 | 27.8 (0.67) | | 102 | 27.7 (0.69) | | 91.3 (7.88) | | 177.5 (16.1) | |
| 4 | 29 | 27.8 (1.41) | | 35 | 27.6 (0.98) | | 114.8 (17.6) | | 172.2 (19.5) | |
| P for heterogeneity |  | **0.003** | |  | 0.35 | | **0.005** | | **0.01** | |
| P for sex-interaction |  |  | |  | 0.83 | |  | | 0.57 | |
|  |  |  | |  |  | |  | |  | |
| BMI (kg/m^2^) categories | 2480 | - | | 2014 | - | |  | |  | |
| <25 | 1005 | - | | 588 | - | | 76.3 (2.88) | | 133.1 (4.50) | |
| 25-29.9 | 861 | - | | 897 | - | | 87.8 (4.21) | | 141.7 (3.23) | |
| ≥ 30 | 614 | - | | 529 | - | | 102.8 (3.09) | | 163.6 (4.92) | |
| P for heterogeneity |  | - | |  | - | | **<0.001** | | **<0.001** | |
| P for sex-interaction |  | - | |  | - | |  | | 0.67 | |
|  |  |  | |  |  | |  | |  | |
| RPM quintiles | 2480 |  | | 2014 |  | | - | | - | |
| 1st (lowest) | 496 | 25.5 (0.23) | | 402 | 26.1 (0.33) | | - | | - | |
| 2nd | 496 | 26.2 (0.25) | | 403 | 27.0 (0.22) | | - | | - | |
| 3rd | 496 | 26.8 (0.36) | | 403 | 27.1 (0.27) | | - | | - | |
| 4h | 496 | 26.9 (0.34) | | 403 | 28.5 (0.77) | | - | | - | |
| 5th (highest) | 496 | 28.4 (0.44) | | 403 | 28.5 (0.30) | | - | | - | |
| P for heterogeneity |  | **<0.001** | |  | **<0.001** | | - | | - | |
| P for sex-interaction |  |  | |  | 0.49 | | - | | - | |
| Abbreviations: BMI, body mass index; PA, physical activity; RPM, red and processed meat; SE, standard error; VLF, vegetable, legume and fruit.  Bolded values are statistically significant. | | | | | | | | | | |
| ^a^ Low leisure-time PA, the lowest VLF consumption quintile, alcohol risk use, or daily smoking. | | | | | | | | | | |
