# Supplementary material for: Meat consumption and obesity: A climate‐friendly way to reduce health inequalities
Source: Public Health Chall. 2024 Mar 15;3(1):e163. doi: 10.1002/puh2.163 (PMC12060756; doi:10.1002/puh2.163)
Supplement: Supplementary file 2 — Table S2 Odds of obesity (BMI ≥ 30 kg/m2) between categories of background variables in women and men in a multivariate modela. [file PUH2-3-e163-s001.docx]

| **Table S2.** Odds of obesity (BMI≥30 kg/m^2^) between categories of background variables in women and in men in a multivariate model^a^. | | | | | | |
| --- | --- | --- | --- | --- | --- | --- |
|  | **Women**  **n=2343** | |  | **Men**  **n=1938** | |  |
|  | **N/n** | **OR (95% CI)** | | **N/n** | **OR (95% CI)** | |
| Age (years) |  |  | |  |  | |
| 18-34 | 55/ 385 | 1 | | 61/288 | 1 | |
| 35-54 | 211/ 869 | **2.06 (1.48-2.86)** | | 163/691 | 1.32 (0.88-1.98) | |
| 55-74 | 307/ 1089 | **1.83 (1.17-2.84)** | | 286/959 | **1.91 (1.30-2.82)** | |
| P for heterogeneity |  | **<0.001** | |  | **0.004** | |
|  |  |  | |  |  | |
| Residential area |  |  | |  |  | |
| Urban areas | 322/ 1417 | 1 | | 267/1126 | 1 | |
| Areas near urban areas, rural centres | 145/ 538 | 1.29 (0.96-1.73) | | 145/481 | **1.60 (1.14-2.25)** | |
| Remote rural areas | 106/ 388 | 1.41 (0.95-2.10) | | 98/331 | 1.18 (0.77-1.81) | |
| P for heterogeneity |  | 0.09 | |  | **0.02** | |
|  |  |  | |  |  | |
| Household structure |  |  | |  |  | |
| Living alone | 136/ 558 | 1 | | 117/379 | 1 | |
| At least one adult and one child | 120/ 646 | **0.61 (0.39-0.96)** | | 103/511 | 1.01 (0.66-1.55) | |
| Adults only | 317/ 1139 | 0.97 (0.72-1.31) | | 290/1048 | 0.98 (0.73-1.32) | |
| P for heterogeneity |  | **0.05** | |  | 0.99 | |
|  |  |  | |  |  | |
| Employment status |  |  | |  |  | |
| Employed | 283/ 1297 | 1 | | 269/1130 | 1 | |
| Other | 290/ 1046 | 1.19 (0.93-1.52) | | 241/808 | 1.18 (0.79-1.76) | |
| P for heterogeneity |  | 0.17 | |  | 0.42 | |
|  |  |  | |  |  | |
| Education |  |  | |  |  | |
| Basic | 103/ 324 | 1 | | 109/303 | 1 | |
| Intermediate | 240/ 882 | 0.79 (0.54-1.16) | | 234/857 | 0.78 (0.54-1.13) | |
| High | 230/ 1137 | **0.64 (0.43-0.94)** | | 167/778 | **0.55 (0.39-0.79)** | |
| P for heterogeneity |  | 0.06 | |  | **0.003** | |
|  |  |  | |  |  | |
| Household income |  |  | |  |  | |
| 1st (lowest) | 104/ 399 | 1 | | 100/305 | 1 | |
| 2nd | 95/ 381 | 1.34 (0.85-2.11) | | 95/385 | **0.58 (0.34-0.97)** | |
| 3rd | 167/ 572 | 1.39 (0.96-2.01) | | 87/377 | **0.60 (0.36-0.99)** | |
| 4th | 111/ 490 | 1.25 (0.86-1.81) | | 112/396 | 0.97 (0.60-1.55) | |
| 5th (highest) | 96/ 501 | 1.05 (0.70-1.58) | | 116/475 | 0.90 (0.57-1.42) | |
| P for heterogeneity |  | 0.30 | |  | **0.03** | |
|  |  |  | |  |  | |
| Leisure-time PA |  |  | |  |  | |
| Moderate or high | 342/1808 | 1 | | 345/1538 | 1 | |
| Low | 231/535 | **2.90 (2.24-3.77)** | | 165/400 | **2.51 (1.95-3.23)** | |
| P for heterogeneity |  | **<0.001** | |  | **<0.001** | |
|  |  |  | |  |  | |
| VLFconsumption quintiles |  |  | |  |  | |
| 2nd – 5th | 451/ 1885 | 1 | | 402/1562 | 1 | |
| 1st (lowest) | 122/458 | 0.84 (0.59-1.21) | | 108/376 | 0.88 (0.66-1.18) | |
| P for heterogeneity |  | 0.36 | |  | 0.40 | |
|  |  |  | |  |  | |
| Alcohol consumption |  |  | |  |  | |
| Moderate or low use | 464/1960 | 1 | | 330/1337 | 1 | |
| Risk use | 109/383 | 1.05 (0.76-1.46) | | 180/601 | 1.23 (0.93-1.64) | |
| P for heterogeneity |  | 0.77 | |  | 0.15 | |
|  |  |  | |  |  | |
| Smoking |  |  | |  |  | |
| Occasional or no | 509/2074 | 1 | | 467/1656 | 1 | |
| Daily | 64/269 | **0.68 (0.48-0.95)** | | 73/282 | **0.61 (0.42-0.89)** | |
| P for heterogeneity |  | **0.02** | |  | **0.01** | |
| Abbreviations: BMI, body mass index; CI, confidence interval; n, individuals in the category; N, obesity cases in the category; OR, odds ratio; PA, physical activity; RPM, red and processed meat; VLF, vegetable, legume and fruit.  Bolded values are statistically significant. | | | | | | |
| ^a^ Model: obesity = RPM consumption, age, energy intake, education, residential area, household income, household structure, employment status, leisure-time PA, VLF consumption, alcohol consumption, and smoking. | | | | | | |
