# Supplementary material for: Meat consumption and obesity: A climate‐friendly way to reduce health inequalities
Source: Public Health Chall. 2024 Mar 15;3(1):e163. doi: 10.1002/puh2.163 (PMC12060756; doi:10.1002/puh2.163)
Supplement: Supplementary file 3 — Table S3 OR (95% CI) of obesity (BMI ≥ 30 kg/m2) between red and processed meat consumption quintiles in women and men in household income quartiles. [file PUH2-3-e163-s004.docx]

| **Table S3.** OR (95% CI) of obesity (BMI≥30 kg/m^2^) between red and processed meat consumption quintiles in women and in men in household income quartiles. | | | | | | | | | | | | | | | | | | |
| --- | --- | --- | --- | --- | --- | --- | --- | --- | --- | --- | --- | --- | --- | --- | --- | --- | --- | --- |
|  | **Women**  **Household income** | | | | | | | |  | **Men**  **Household income** | | | | | | | |  |
|  | **1st Q**  **n=506** | |  | **2nd-3rd Q**  **n=1225** | |  | **4th Q**  **n=661** | |  | **1st Q**  **n=396** | |  | **2nd-3rd Q**  **n=1031** | |  | **4th Q**  **n=541** | |  |
|  | **N/n** | **OR (95% CI)** | | **N/n** | **OR (95% CI)** | | **N/n** | **OR (95% CI)** | | **N/n** | **OR (95% CI)** | | **N/n** | **OR (95% CI)** | | **N/n** | **OR (95% CI)** | |
| Adjustment model 1^a^ |  |  | |  |  | |  |  | |  |  | |  |  | |  |  | |
| RPM quintiles |  |  | |  |  | |  |  | |  |  | |  |  | |  |  | |
| 1st (lowest), reference | 18/93 | 1 | | 42/236 | 1 | | 23/155 | 1 | | 19/74 | 1 | | 35/207 | 1 | | 16/109 | 1 | |
| 2nd | 16/89 | 0.74 (0.34-1.61) | | 50/244 | 1.03 (0.67-1.60) | | 25/139 | 1.51 (0.78-2.95) | | 21/81 | 0.92 (0.42-2.00) | | 45/205 | 1.41 (0.83-2.40) | | 22/111 | 1.40 (0.58-3.35) | |
| 3rd | 24/107 | 1.21 (0.51-2.86) | | 73/250 | **2.16 (1.35-3.46)** | | 28/129 | 1.62 (0.87-3.02) | | 18/68 | 0.83 (0.36-1.90) | | 47/218 | 1.33 (0.76-2.34) | | 26/109 | 1.71 (0.71-4.16) | |
| 4h | 24/91 | 1.55 (0.71-3.38) | | 80/266 | **2.06 (1.25-3.41)** | | 26/126 | 1.94 (0.93-4.04) | | 27/92 | 1.83 (0.77-4.37) | | 63/204 | **2.73 (1.59-4.71)** | | 36/103 | **2.88 (1.28-6.48)** | |
| 5th (highest) | 47/126 | **3.86 (1.60-9.34)** | | 85/229 | **2.99 (1.85-4.82)** | | 26/112 | **3.04 (1.55-5.95)** | | 33/81 | 2.23 (0.94-5.34) | | 72/197 | **3.01 (1.82-5.00)** | | 36/109 | **2.95 (1.31-6.66)** | |
| P for heterogeneity |  | **0.01** | |  | **<0.001** | |  | **0.03** | |  | **0.04** | |  | **<0.001** | |  | **0.005** | |
| P for income interaction |  |  | |  |  | |  | 0.68 | |  |  | |  |  | |  | 0.97 | |
| P for income-sex interaction |  |  | |  |  | |  |  | |  |  | |  |  | |  | 0.81 | |
|  |  |  | |  |  | |  |  | |  |  | |  |  | |  |  | |
| Adjustment model 2 ^b^ |  |  | |  |  | |  |  | |  |  | |  |  | |  |  | |
| RPM quintiles |  |  | |  |  | |  |  | |  |  | |  |  | |  |  | |
| 1st (lowest), reference | 18/92 | 1 | | 42/236 | 1 | | 23/155 | 1 | | 19/73 | 1 | | 35/207 | 1 | | 16/109 | 1 | |
| 2nd | 16/89 | 0.70 (0.31-1.59) | | 49/243 | 1.00 (0.64-1.58) | | 25/139 | 1.59 (0.83-3.05) | | 21/81 | 0.79 (0.35-1.79) | | 45/205 | 1.40 (0.83-2.39) | | 22/111 | 1.34 (0.52-3.43) | |
| 3rd | 24/107 | 1.20 (0.49-2.92) | | 73/250 | **2.27 (1.42-3.62)** | | 28/129 | 1.66 (0.88-3.12) | | 17/67 | 0.65 (0.25-1.70) | | 47/217 | 1.35 (0.77-2.39) | | 26/109 | 1.65 (0.66-4.12) | |
| 4h | 24/90 | 1.64 (0.69-3.89) | | 80/266 | **2.11 (1.27-3.50)** | | 26/126 | 1.98 (0.88-4.48) | | 27/92 | 1.60 (0.72-3.58) | | 63/204 | **2.70 (1.56-4.70)** | | 36/103 | **2.69 (1.21-5.97)** | |
| 5th (highest) | 47/126 | **3.93 (1.72-8.96)** | | 85/229 | **2.94 (1.77-4.86)** | | 26/112 | **2.89 (1.43-5.81)** | | 33/81 | 1.75 (0.73-4.17) | | 72/197 | **2.98 (1.77-5.01)** | | 36/109 | **2.30 (1.00-5.31)** | |
| P for heterogeneity |  | **0.003** | |  | **<0.001** | |  | 0.07 | |  | 0.08 | |  | **<0.001** | |  | **0.03** | |
| P for income interaction |  |  | |  |  | |  | 0.49 | |  |  | |  |  | |  | 0.95 | |
| P for income-sex interaction |  |  | |  |  | |  |  | |  |  | |  |  | |  | 0.67 | |
|  |  |  | |  |  | |  |  | |  |  | |  |  | |  |  | |
| Adjustment model 3 ^c^ |  |  | |  |  | |  |  | |  |  | |  |  | |  |  | |
| RPM quintiles |  |  | |  |  | |  |  | |  |  | |  |  | |  |  | |
| 1st (lowest), reference | 17/88 | 1 | | 40/229 | 1 | | 22/153 | 1 | | 18/69 | 1 | | 35/206 | 1 | | 16/109 | 1 | |
| 2nd | 16/86 | 0.94 (0.41-2.16) | | 47/240 | 1.00 (0.62-1.61) | | 25/136 | 1.52 (0.76-3.02) | | 21/81 | 0.88 (0.37-2.05) | | 45/203 | 1.38 (0.81-2.36) | | 22/111 | 1.33 (0.52-3.40) | |
| 3rd | 23/105 | 1.55 (0.61-3.93) | | 73/247 | **2.28 (1.36-3.80)** | | 28/123 | **1.89 (1.00-3.57)** | | 17/66 | 0.80 (0.30-2.08) | | 47/214 | 1.34 (0.76-2.38) | | 26/108 | 1.72 (0.67-4.44) | |
| 4h | 22/87 | 1.84 (0.69-4.91) | | 80/264 | **2.07 (1.22-3.51)** | | 26/125 | 1.84 (0.82-4.11) | | 27/91 | 2.00 (0.84-4.74) | | 63/204 | **2.66 (1.54-4.60)** | | 36/103 | **2.57 (1.14-5.80)** | |
| 5th (highest) | 46/125 | **5.09 (1.97-13.2)** | | 83/226 | **2.52 (1.46-4.35)** | | 26/112 | **2.77 (1.40-5.50)** | | 33/81 | 2.03 (0.84-4.87) | | 69/191 | **2.96 (1.75-5.01)** | | 36/109 | 2.15 (0.91-5.06) | |
| P for heterogeneity |  | **0.005** | |  | **<0.001** | |  | 0.07 | |  | **0.05** | |  | **<0.001** | |  | 0.07 | |
| P for income interaction |  |  | |  |  | |  | 0.63 | |  |  | |  |  | |  | 0.96 | |
| P for income-sex interaction |  |  | |  |  | |  |  | |  |  | |  |  | |  | 0.71 | |
| Abbreviations: BMI, body mass index; CI, confidence interval; n, individuals in the category; N, obesity cases in the category; OR, odds ratio; Q, quartile; RPM, red and processed meat.  Bolded values are statistically significant. | | | | | | | | | | | | | | | | | | |
| ^a^ Adjusted for age and energy intake. | | | | | | | | | | | | | | | | | | |
| ^b^ Adjusted for age, energy intake, residential area, education, household structure and employment status. | | | | | | | | | | | | | | | | | | |
| ^c^ Adjusted for age, energy intake, residential area, education, household structure, employment status, leisure-time physical activity (PA), vegetable, legume and fruit (VLF) consumption, alcohol consumption, and smoking. | | | | | | | | | | | | | | | | | | |
