# Supplementary material for: Meat consumption and obesity: A climate‐friendly way to reduce health inequalities
Source: Public Health Chall. 2024 Mar 15;3(1):e163. doi: 10.1002/puh2.163 (PMC12060756; doi:10.1002/puh2.163)
Supplement: Supplementary file 4 — Table S4 Odds of obesity (BMI ≥ 30 kg/m2) between red and processed meat consumption tertiles in interaction categories of education and unfavourable lifestyle habits (n = 4281). [file PUH2-3-e163-s002.docx]

| **Table S4.** Odds of obesity (BMI≥30 kg/m^2^) between red and processed meat consumption tertiles in interaction categories of education and unfavorable lifestyle habits (n=4281). | | | | | | | | | | |
| --- | --- | --- | --- | --- | --- | --- | --- | --- | --- | --- |
|  |  | **RPM tertiles (g)** | | | | | | | |  |
|  |  | **1st T**  **n=849** | |  | **2nd T**  **n=2591** | |  | **3rd T**  **n=841** | |  |
| **Education** | **Lifestyle composite variable^a^** | **N/n** | **OR (ref.)** | | **N/n** | **OR (95% CI)** ^b^ | | **N/n** | **OR (95% CI)** ^b^ | |
| Basic | 0 unfavorable habits | 21/104 | 1 | | 27/95 | 1.21 (0.59-2.50) | | 24/75 | 1.46 (0.65-3.26) | |
|  | 1 unfavorable habit | 18/50 | 1 | | 20/63 | 0.96 (0.36-2.60) | | 34/71 | 1.88 (0.82-4.29) | |
|  | 2-4 unfavorable habits | 15/37 | 1 | | 13/48 | 0.37 (0.13-1.04) | | 40/84 | 1.47 (0.54-4.02) | |
|  | P for lifestyle-interaction |  |  | |  |  | |  | 0.13 | |
|  |  |  |  | |  |  | |  |  | |
| Intermediate | 0 unfavorable habits | 47/274 | 1 | | 56/263 | **1.98 (1.08-3.65)** | | 66/218 | **2.25 (1.38-3.66)** | |
|  | 1 unfavorable habit | 47/178 | 1 | | 48/199 | 0.80 (0.47-1.37) | | 74/226 | **2.05 (1.17-3.59)** | |
|  | 2-4 unfavorable habits | 19/79 | 1 | | 34/118 | 1.38 (0.75-2.56) | | 83/184 | **3.36 (1.77-6.38)** | |
|  | P for lifestyle-interaction |  |  | |  |  | |  | 0.16 | |
|  |  |  |  | |  |  | |  |  | |
| High | 0 unfavorable habits | 46/421 | 1 | | 66/377 | **2.06 (1.35-3.12)** | | 56/269 | **2.30 (1.40-3.78)** | |
|  | 1 unfavorable habit | 37/196 | 1 | | 63/202 | **2.07 (1.29-3.31)** | | 51/194 | **2.18 (1.23-3.86)** | |
|  | 2-4 unfavorable habits | 19/81 | 1 | | 19/71 | 0.91 (0.37-2.25) | | 40/104 | 1.75 (0.77-3.97) | |
|  | P for lifestyle-interaction |  |  | |  |  | |  | 0.53 | |
|  | P for education-lifestyle-interaction |  |  | |  |  | |  | 0.15 | |
| Abbreviations: BMI, body mass index; CI, confidence interval; n, individuals in the category; N, obesity cases in the category; OR, odds ratio; Q, quintile; ref., reference; RPM, red and processed meat; T, tertile.  Bolded values are statistically significant. | | | | | | | | | | |
| ^a^ Low leisure-time physical activity (PA), the lowest vegetable, legume and fruit (VLF) consumption quintile, alcohol risk use, or daily smoking. | | | | | | | | | | |
| ^b^ Adjusted for sex, age, energy intake, residential area, household income, household structure and employment status. | | | | | | | | | | |
